# The Complete Chloroplast Genomes of Six *Ipomoea* Species and Indel Marker Development for the Discrimination of Authentic Pharbitidis Semen (Seeds of *I. nil* or *I. purpurea*)

**DOI:** 10.3389/fpls.2018.00965

**Published:** 2018-07-05

**Authors:** Inkyu Park, Sungyu Yang, Wook J. Kim, Pureum Noh, Hyun O. Lee, Byeong C. Moon

**Affiliations:** ^1^K-herb Research Center, Korea Institute of Oriental Medicine, Daejeon, South Korea; ^2^Phyzen Genomics Institute, Seongnam, South Korea

**Keywords:** Ipomoeeae, plastid genome, divergent region, phylogenetic relationship, indel marker

## Abstract

*Ipomoea* L. is the largest genus within the Convolvulaceae and contains 600–700 species. *Ipomoea* species (morning glories) are economically valuable as horticultural species and scientifically valuable as ecological model plants to investigate mating systems, molecular evolution, and both plant–herbivore and plant–parasite interactions. Furthermore, the dried seeds of *I. nil* or *I. purpurea* are used in Korean traditional herbal medicines. In this study, chloroplast (cp) genomes were sequenced from six *Ipomoea* species, namely, *I. nil* and *I. purpurea* and, for the first time, *I. triloba, I. lacunosa, I. hederacea*, and *I. hederacea* var. *integriuscula*. The cp genomes were 161,354–161,750 bp in length and exhibited conserved quadripartite structures. In total, 112 genes were identified, including 78 protein-coding regions, 30 transfer RNA genes, and 4 ribosomal RNA genes. The gene order, content, and orientation of the six *Ipomoea* cp genomes were highly conserved and were consistent with the general structure of angiosperm cp genomes. Comparison of the six *Ipomoea* cp genomes revealed locally divergent regions, mainly within intergenic spacer regions (*petN-psbM, trnI-CAU-ycf2, ndhH-ndhF, psbC-trnS*, and *ccsA-ndhD*). In addition, the protein-coding genes *accD, cemA*, and *ycf2* exhibited high sequence variability and were under positive selection (Ka/Ks > 1), indicating adaptive evolution to the environment within the *Ipomoea* genus. Phylogenetic analysis of the six *Ipomoea* species revealed that these species clustered according to the APG IV system. In particular, *I. nil* and *I. hederacea* had monophyletic positions, with *I. purpurea* as a sister. *I. triloba* and *I. lacunosa* in the section Batatas and *I. hederacea* and *I. hederacea* var. *integriuscula* in the section Quamoclit were supported in this study with strong bootstrap values and posterior probabilities. We uncovered high-resolution phylogenetic relationships between Ipomoeeae. Finally, indel markers (IPOTY and IPOYCF) were developed for the discrimination of the important herbal medicine species *I. nil* and *I. purpurea*. The cp genomes and analyses in this study provide useful information for taxonomic, phylogenetic, and evolutionary analysis of the *Ipomoea* genome, and the indel markers will be useful for authentication of herbal medicines.

## Introduction

Chloroplasts (cp) are among the most important organelles in plants, having important roles in photosynthesis and carbon fixation as well as in the biosynthesis of starch, fatty acids, amino acids, and pigments (Jansen and Ruhlman, 2012; Daniell et al., 2016). Cp genomes in higher plants are 120–180 kb and, in general, exhibit a quadripartite structure consisting of two single-copy regions, namely, the large single-copy (LSC) and small single-copy (SSC), as well as two copies of a larger inverted repeat (IR) region. Angiosperm cp genomes generally contain 80 protein-coding genes, 4 ribosomal RNA (rRNA) genes, and 30 transfer RNA (tRNA) genes (Wicke et al., 2011). While the majority of cp genomes exhibit highly conserved structures, some reveal structural variations, IR loss, and gene loss as a result of adaptation to their environments (Delannoy et al., 2011; Wicke et al., 2013). Next-generation technologies have allowed the rapid sequencing of many cp genomes in recent years. These abundant cp genomes have facilitated the verification of evolutionary relationships and allowed detailed phylogenetic classifications to be conducted at group, family, and even genus level in Plantae (Jansen et al., 2007; Parks et al., 2009). Furthermore, cp genomes can be used for species identification through the use of DNA barcodes and molecular markers that allow morphologically similar species to be distinguished (Kim et al., 2015; Park et al., 2017a,b). Thus, cp genomes can be used for practical applications such as species identification as well as for fundamental research into biological processes and evolutionary relationships.

*Ipomoea* is the largest genus in the Convolvulaceae family, with 600–700 species (Austin and Huáman, 1996; Wilkin, 1999). *Ipomoea* species are widely distributed across tropical, subtropical, and some temperate regions worldwide (Austin and Huáman, 1996; Wilkin, 1999). *I. nil, I. purpurea, I. tricolor*, and *I. batatas* are particularly well-known *Ipomoea* species. *I. nil* exhibited spontaneous mutations related to floricultural traits. These mutants have been exploited as ornamental plants in horticulture (Hoshino et al., 2016). *Ipomoea* is an emerging model system for ecological genomics studies (Baucom et al., 2011; Eserman et al., 2014). Ecological studies of *Ipomoea* have answered many diverse questions about the *Ipomoea* mating system, the evolution of floral color pathways, and both plant–herbivore and plant–parasite interactions (Baucom et al., 2011). Visitations by natural pollinators and the selfing rate in various *Ipomoea* species vary in proportion to the number of offspring derived from self-fertilization. These findings are exemplified by extreme differences in floral color in *Ipomoea*, which ranges from white or yellow to red or purple (Ennos and Clegg, 1983; Epperson and Clegg, 1992; Baucom et al., 2011). These flower colors (which are associated with anthocyanin pigments) have evolved *via* parallel evolution due to various factors, such as enzyme-coding genes (F3′H) or regulatory modifications (Des Marais and Rausher, 2008; Streisfeld and Rausher, 2009). Furthermore, *I. purpurea* and *I. hederacea* are model plants used to study plant–herbivore interactions based on ecological evolution (Tiffin and Rausher, 1999; Baucom et al., 2011). Several studies show that insects have affected natural selection for plant resistance in *Ipomoea* and the tradeoff between resistance and tolerance in plant defense responses. In addition, studies of plant–herbivore interactions point to the coevolution between *I. purpurea* and *I. hederacea* and their competitors (Rausher and Fry, 1993; Simonsen and Stinchcombe, 2007). Studies examining the evolution of *Ipomoea* in response to plant pathogens show that quantitative resistance to *Colletotrichum dematium* is genetically correlated to quantitative resistance to an insect herbivore and that an oomycete exhibits host specialization in *Ipomoea* (Simms and Rausher, 1993; Sato et al., 2009). Therefore, *Ipomoea* species represent highly important resources that have contributed strongly to ecological studies.

In Korean traditional medicine, the dried seeds of *I. nil* or *I. purpurea* are an important herbal medicine, namely, Pharbitidis Semen, which is used to eliminate toxins or heat, as a diuretic, and as a treatment for constipation relief treatment [Korea Institute of Oriental Medicine (KIOM), 2016]. Pharbitidis Semen is designated as a medicine in Korea, and is regulated by the Ministry of Food and Drug Safety due to its pharmaceutical activity and potential toxicity (Korean Food Standard Codex, 2010). Only seeds of *I. nil* or *I. purpurea* are considered to be authentic Pharbitidis Semen. In general, *Ipomoea* seeds are trigonous and are brown to dark-brown in color (McDonald, 1995), and seeds from different *Ipomoea* species are morphologically similar and difficult to distinguish with the unaided eye. As a result, Pharbitidis Semen in Korean and Chinese herbal markets often contains a mixture of seeds from *I. nil* and *I. purpurea* and seeds from other *Ipomoea* species. Indiscriminate use of these adulterated Pharbitidis Semen preparations could cause unforeseen side-effects and threaten its use as a safe and reliable medication. Methods are therefore needed to distinguish good quality Pharbitidis Semen preparations from adulterated preparations.

Molecular tools can be used for accurate species identification and authentication of herbal medicine. In particular, the universal DNA barcode markers ITS, *matK*, and *rbcL* are widely used for species classification and phylogenetic analysis in Plantae (Semagn et al., 2006; Sucher and Carles, 2008; Chen et al., 2010; Hollingsworth et al., 2011). These barcode markers offer rapid and accurate species identification from short DNA sequences. However, some plants, particularly closely related species, cannot be readily distinguished using these markers. The cp genome has emerged as an alternative to DNA barcoding markers for species identification and phylogenetic studies. Comparison of cp genomes highlighted several variable regions that could be used for the development of markers to allow species discrimination (Kim et al., 2015; Park et al., 2017a). While the cp genome was generally more highly conserved than the nuclear genome, abundant genetic variations such as insertion/deletions (indels) and single nucleotide polymorphisms (SNPs) were identified between species. Several studies developed cp markers for identification of closely related species, including indel and SNP markers for *Panax ginseng* subspecies (Kim et al., 2015), and indel tandem repeat copy number variation markers for *Fagopyrum tataicum* and *F. esculentum* (Cho et al., 2015). In another example, sequence characterized amplified region markers were developed to resolve *Aconitum* species. Two indel markers derived from large variable regions were used to distinguish three *Aconitum* species, *A. pseudolaeve, A. longecassidatum*, and *A. barbatum*, and a small species-specific 6 bp insertion was used to distinguish *A. coreanum* (Park et al., 2017a,b). *Chenopodium quinoa* and *C. album* were distinguished using indel tandem repeat copy number variation markers (Hong et al., 2017). These examples illustrate the utility of the cp genome for plant species identification and for the authentication and identification of herbal medicines.

Previous phylogenetic analysis of the genus *Ipomoea* was unclear, with unresolved monophyly at the subgenera level (Manos et al., 2001; Stefanoviæ et al., 2002). Previously, molecular phylogenetic relationships within the Convolvulaceae were evaluated using ITS and four cp loci (Stefanoviæ et al., 2002; Miller et al., 2004), but this analysis identified only monophyletic or weak relationships in tribe Ipomoeeae. A separate analysis of four cp loci divided tribe Ipomoeeae into two clades, Astripomoeinae and Argyreiinae, but morphological features were not considered (Stefanoviæ et al., 2003). Recently, Eserman et al. (2014), described high-resolution phylogenetic relationships in tribe Ipomoeeae and the Astripomoeinae and Argyreiinae clades and identified similar divergence times (23–26 MYA) based on whole cp genomes. Analysis of 32 cp genomes from magnoliids, monocots, and eudicots verified phylogenetic relationships for sweet potato (*I. batatas*) (Yan et al., 2015). Analysis of the completed nuclear genome identified a whole-genome duplication event in *I. nil* and showed divergence from Solanaceae at 75.25 MYA (Hoshino et al., 2016). Examination of *Ipomoea*, which contains hundreds of species, identified a range of useful genomic information, but this was not sufficient for high-resolution determination of phylogenetic relationships in Ipomoeeae. Further research is needed to understand the evolutionary relationships within tribe Ipomoeeae as well as the *Ipomoea* genus.

Here, samples of herbal medicine species *I. nil* and *I. purpurea* and four closely related *Ipomoea* species with similar seed structures were collected and their cp genomes were compared. This study aimed to (1) characterize six *Ipomoea* cp genomes and identify genetically variable regions by comparison of their global structures, (2) develop novel molecular markers for use in authentication of herbal medicine species, and (3) understand evolutionary relationships within tribe Ipomoeeae through enhanced phylogenic studies in conjunction with previously reported cp genomes.

## Materials and Methods

### Plant Materials

Fresh leaves of six *Ipomoea* species were collected from native habitats in Korea and used for cp genome sequencing. *I. nil, I. purpurea, I. hederacea, I. hederacea* var. *integriuscula, I. lacunosa*, and *I. triloba* were assigned identification numbers, and specimens were registered in the Korean Herbarium of Standard Herbal Resources (Index Herbariorum code KIOM) at the KIOM. The plant samples used for cp genome analysis and indel validation in this study are listed in Supplementary Table S1.

### Genome Sequencing and Assembly

DNA was extracted using a DNeasy Plant Maxi Kit (Qiagen, Valencia, CA, United States) according to the manufacturer’s instructions. Illumina short-insert paired-end sequencing libraries were constructed and generated using the NextSeq platform (Illumina, San Diego, CA, United States). *De novo* assembly was used to construct cp genomes from low-coverage whole-genome sequences. Trimmed paired-end reads (Phred scores ≥20) were assembled using CLC genome assembler (ver. 4.06 beta, CLC Inc., Aarhus, Denmark) with default parameters. SOAP *de novo* gap closer was used to fill gaps based on alignment of paired-end reads (Luo et al., 2012). Principal contigs representing the cp genome were retrieved from total contigs using Nucmer (Delcher et al., 2003), and aligned contigs were ordered using the cp genome sequence of *I. nil* (AP017304) as a reference (Hoshino et al., 2016).

### Genome Annotation and Comparative Analysis

Gene annotation of the six *Ipomoea* cp genomes was performed using GeSeq (Tillich et al., 2017), and the annotation results were concatenated using an in-house script pipeline. Protein-coding sequences were manually curated and confirmed using Artemis (Carver et al., 2008), and checked against the NCBI protein database. The tRNAs were confirmed with tRNAscan-SE 1.21 (Lowe and Eddy, 1997). IR region sequences were confirmed using IR finder and RepEx (Warburton et al., 2004; Gurusaran et al., 2013). Circular maps of the six *Ipomoea* cp genomes were obtained using OGDRAW (Lohse et al., 2007). GC content and relative synonymous codon usages (RSCU) were analyzed using MEGA6 software (Tamura et al., 2013). The mVISTA program in Shuffle-LAGAN mode was used to compare the six *Ipomoea* cp genomes using the *I. nil* cp genome as a reference. DnaSP version 5.1 (Librado and Rozas, 2009) was used to calculate nucleotide variability (Pi) among the six *Ipomoea* cp genomes. Substitution rates Ka and Ks were estimated with PAL2NAL (Suyama et al., 2006). LSC/IR, IR/SSC, SSC/IR, and IR/LSC regions of completed cp genomes were validated using PCR-based sequencing. Primer information and sequence alignment results are listed in Supplementary Tables S2, S3.

### Repeat Analysis

SSRs in six *Ipomoea* cp genomes were detected using MISA (Thiel, 2003) with the minimum number of repeat parameters set to 10, 5, 4, 3, 3, and 3 for mono-, di-, tri-, tetra-, penta-, and hexa-nucleotides, respectively. Tandem repeats were ≥20 bp with minimum alignment score and maximum period size of 50 and 500, respectively, and the identity of repeats was set to ≥90% (Benson, 1999).

### Phylogenetic Analysis

A total of 38 cp genomes, including 36 from Convolvulaceae, were used for phylogenetic analyses, along with *Nicotiana tabacum* (GenBank acc. NC_001879.2) and *Capsicum annuum* var. *glabriusculum* (GenBank acc. KJ619462.1) as outgroups. Of these, 32 cp genome sequences were downloaded from the NCBI GenBank (Supplementary Table S4). MAFFT (Katoh et al., 2002) was used to construct molecular phylogenetic trees from alignments of 48 conserved protein-coding genes, and the sequences were manually adjusted using Bioedit (Hall, 1999). The best-fitting model of nucleotide substitutions was determined using Akaike Information Criterion in JModeltest V2.1.10 (Darriba et al., 2012). The GTR + I + G model was used in both. Maximum likelihood (ML) analysis was performed using RaxML v 8.0.5 (Stamatakis, 2014) with 1000 bootstrap replicates. Bayesian Inference (BI) analysis was performed using MrBayes 3.2.2 (Ronquist et al., 2012) with two-independent runs and four chains using Markov Chain Monte Carlo run simultaneously for one million generations. Trees were sampled every 5,000,000 generations, with the first 25% discarded as burn-in. Trees were determined from 50% majority-rule consensus trees to estimate posterior probabilities (PP). The reconstructed trees were visualized using Fig tree V.1.4.2 (Rambaut, 2012).

### InDel Marker Development and Validation for *I. nil* and *I. purpurea*

Indel regions were detected by the alignment of six *Ipomoea* cp genome sequences and comparison of mVISTA similarities. Primers for indel markers were designed using NCBI Primer-BLAST. Specificity of indel markers was confirmed using PCR amplification with 20 ng of genomic DNA extracted from 23 samples of 6 *Ipomoea* species in a 20 μl PCR mixture with 10 pmol of IPOTY or IPOYCF indel primers. Amplification of both IPOTY and IPOYCF was conducted on a Pro Flex PCR system (Applied Biosystems, Waltham, MA, United States) with the following amplification parameters: initial denaturation at 95°C for 2 min; 35 cycles at 95°C for 50 s, 62°C for 50 s, and 72°C for 50 s; and final extension at 72°C for 5 min. PCR products were separated on a 2% agarose gel for 40 min at 150V. DNA fragments were extracted from agarose using a Gel Extraction Kit, subcloned into the pGEM-T Easy vector (Promega, WI, United States), and sequenced on a DNA sequence analyzer (ABI 3730, Applied Biosystems Inc., CA, United States). The six *Ipomoea* species germplasms used are listed in Supplementary Table S1.

## Results and Discussion

### Chloroplast Genome Organization of Six *Ipomoea* Species

**FIGURE 1:**
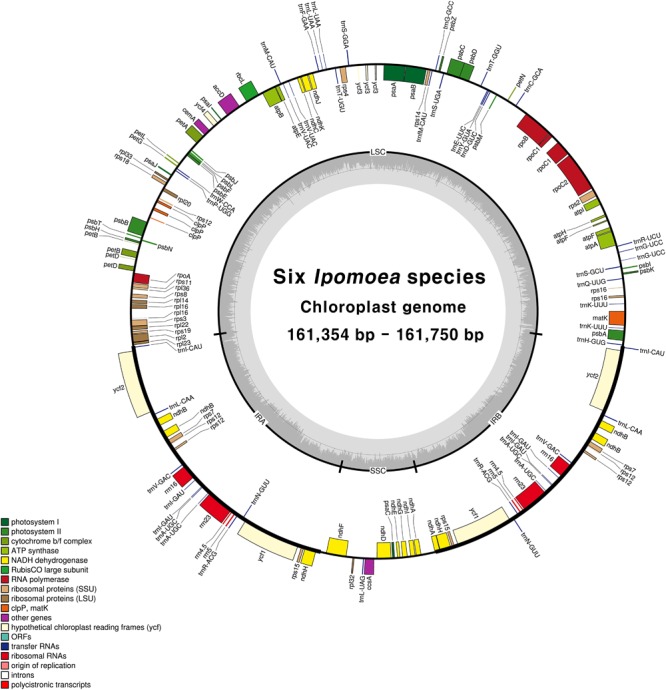
Circular gene map of chloroplast genomes from six *Ipomoea* species. Genes drawn inside the circle are transcribed clockwise, and those outside the circle are transcribed counterclockwise. The darker gray in the inner circle represents GC content. The gene map corresponds to *I. nil* chloroplast genome.

Illumina sequencing generated 1.3–1.6 Gb of trimmed paired-end reads from six *Ipomoea* species (Supplementary Table S5). The six species yielded complete circular chloroplast cp genomes of 161,354–161,750 bp, with 384–611× coverage (Supplementary Table S6). As in most land plants, the *Ipomoea* cp genomes exhibited quadripartite structures consisting of a pair of IRs (61,220–62,122 bp) separated by LSC (87,579–88,134 bp) and SSC (12,039–12,101 bp) regions (**Figure 1** and **Table 1**). The six *Ipomoea* cp genomes had similar GC contents, with higher GC contents observed in the IR regions (41%) than in the single-copy regions (LSC, 36% and SSC, 32%), consistent with previously reported cp genomes (Eserman et al., 2014; Yan et al., 2015). The gene content, order, and orientation were similar in the six *Ipomoea* cp genomes. The 112 unique genes consisted of 78 protein-coding genes and 30 tRNAs, with 17 duplicated genes in the 6 genomes (**Table 2**). Seventeen of the genes contained introns, fourteen with a single intron and two (*ycf3* and *clpP*) with two introns (Supplementary Table S7). The genes *psbL* and *ndhD* had the alternative start codon ACG, and *rps19* started with GTG. Use of ACG and GTG as start codons is common for several genes in the cp genomes of land plants (Sasaki et al., 2003; Kahlau et al., 2006; Gao et al., 2009; Sanchez-Puerta and Abbona, 2014). The codon usage and anticodon recognition patterns of the six *Ipomoea* cp genomes are shown in Supplementary Figure S1A. Protein-coding genes comprised 28,280 codons in *I. hederacea* to 28,434 codons in *I. triloba*, which was consistent with other plant cp genomes. Codons for leucine, isoleucine, and serine were the most abundant, whereas those for cysteine and tryptophan were found least often (Supplementary Figure S1B). RSCU values revealed synonymous codon usage bias, with a high proportion of synonymous codons having A or T in the third position. As expected, codons for arginine, leucine, and serine had abundant synonymous codons and higher RSCU values due to their importance as components of cp genes related to biosynthetic processes (Wang et al., 2016). The RSCU values of the six *Ipomoea* cp genomes were consistent with those of other higher plants. This phenomenon is indicative of stable cp evolution, which protects important cp genes against harmful mutations and adaptive selective pressures in important cp genes (Wang et al., 2016; Ivanova et al., 2017; Zuo et al., 2017). The six *Ipomoea* genomes exhibited typical features of *Ipomoea* cp genomes and had similar genome structures, gene orders, and gene contents, including introns and base composition, to one another.

**Table 1:** Features of six *Ipomoea* chloroplast genomes.

Species	*I. nil*	*I. purpurea*	*I. hederacea*	*I. hederacea* var. *integriuscula*	*I. lacunosa*	*I. triloba*
Accession number	MG973745	MG973746	MG973747	MG973748	MG973749	MG973750
Total cp genome size (bp)	161,747	161,629	161,354	161,714	161,492	161,750
Large single-copy (LSC) region (bp)	88,097	88,134	88,041	88,083	87,579	87,589
Inverted repeat (IR) region (bp)	61,564	61,394	61,220	61,538	61,862	62,122
Small single-copy (SSC) region (bp)	12,086	12,101	12,093	12,093	12,051	12,039
Total number of genes (unique)	112	112	112	112	112	112
Protein-coding gene (unique)	78	78	78	78	78	78
rRNA (unique)	4	4	4	4	4	4
tRNA (unique)	30	30	30	30	30	30
GC content (%)	38	38	38	38	38	38
LSC (%)	36	36	36	36	36	36
IR (%)	41	41	41	41	41	41
SSC (%)	32	32	32	32	32	32

**Table 2:** Genes in the chloroplast genomes of six *Ipomoea* species.

Gene groups	Gene names
Photosystem I	*psaA, B, C, I, J, ycf3*^2^, *ycf4*
Photosystem II	*psbA, B, C, D, E, F, H, I, J, K, L, M, N, T, Z*
Cytochrome b6/f	*petA, B*^1^, *D*^1^, *G, L, N*
ATP synthase	*atpA, B, E, F*^1^, *H, I*
Rubisco	*rbcL*
NADH oxidoreductase	*ndhA*^1^, *B*^1,3^, *C, D, E, F, G, H*^3^, *I, J, K*
Large subunit ribosomal proteins	*rpl2, 14, 16*^1^, *20, 22, 23, 32, 33, 36*
Small subunit ribosomal proteins	*rps2, 3, 4, 7*^3^, *8, 11, 12* ^2-4^, *14, 15*^3^, *16*^1^, *18, 19*
RNA polymerase	*rpoA, B, C1*^1^, *C2*
Unknown function protein-coding gene	*ycf1*^3^, *2*^3^
Other genes	*accD, ccsA, cemA, clpP*^2^, *matK*
Ribosomal RNAs	*rrn16*^3^, *23*^3^, *4.5*^3^, *5*^3^
Transfer RNAs	*trnA-UGC*^1,3^, *trnC-GCA, trnD-GUC, trnE-UUC, trnF-GAA, trnfM-CAU, trnG-GCC, trnG-UCC*^1^, *trnH-GUG, trnI-CAU*^3^, *trnI-GAU*^1,3^, *trnK-UUU*^1^, *trnL-CAA*^3^, *trnL-UAA, trnL-UAG, trnM-CAU, trnN-GUU*^3^, *trnP-UGG, trnQ-UUG, trnR-ACG*^3^, *trnR-UCU, trnS-GCU, trnS-GGA, trnS-UGA, trnT-GGU, trnT-UGU, trnV-GAC, trnV-UAC, trnW-CCA, trnY-GUA*

*^*1*^Gene containing a single intron; ^*2*^Gene containing two introns; ^*3*^Two gene copies in IRs; ^*4*^Trans-splicing gene.*

### SSR and Tandem Repeat Analysis in Six *Ipomoea* Chloroplast Genomes

**FIGURE 2:**
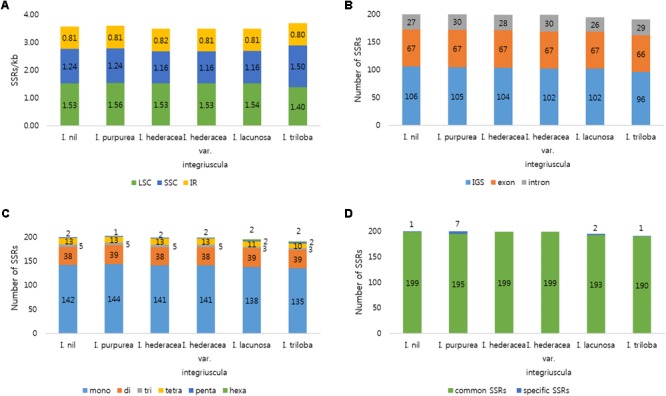
Distribution of SSRs in the six *Ipomoea* chloroplast genomes. **(A)** Number of SSRs per unit length in genomic regions. **(B)** Distribution of SSRs in intergenic spacer (IGS), exon, and intron regions. **(C)** Distribution of SSR types. **(D)** Number of common and species-specific SSRs among the six *Ipomoea* chloroplast genomes.

**FIGURE 3:**
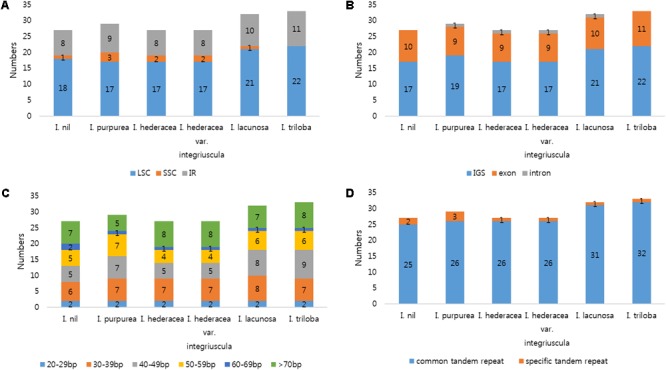
Analysis of tandem repeats in six *Ipomoea* chloroplast genomes. **(A)** Distribution of tandem repeats in genomic regions. **(B)** Distribution of tandem repeats in intergenic spacer (IGS), exon, and intron regions. **(C)** Distribution and lengths of tandem repeats. **(D)** Number of common and species-specific tandem repeats among the six *Ipomoea* chloroplast genomes.

SSRs (1–6 nucleotide repeats) were distributed abundantly across the cp genome. SSRs from cp genomes can be used for analysis of phylogenetic relationships and population genetics due to their high polymorphism rates and stable reproducibility (Powell et al., 1995; Dong et al., 2013; Yang et al., 2016). Most SSRs contained A or T units, contributing to the overall AT richness of the cp genome (Qian et al., 2013). Here, MISA software identified 191–202 SSRs in the six *Ipomoea* cp genomes. Most SSRs were found in single-copy regions (LSC and SSC) and non-coding regions (**Figure 2**). Mononucleotide motifs were the most abundant repeat type, with dinucleotide motifs, the second most abundant in the six *Ipomoea* cp genomes (**Figure 2C**). SSRs were compared between the genomes to identify common and species-specific SSRs. *I. purpurea* contained seven specific SSRs, whereas no *I. hederacea*-specific SSRs were identified. The *Ipomoea* cp SSRs encompassed abundant variation and will be useful genomic resources for marker development and population genetic studies of *Ipomoea* species. Tandem repeat sequences influence genome structure with respect to genome size, genome rearrangement, and gene duplication (Nie et al., 2012). Here, 27–33 tandem repeats of >20 bp were identified in the six *Ipomoea* genomes, averaging 56–74 bp in length (**Figure 3**). Of these, most were located in non-coding LSC and SSC regions (**Figure 3A**). On average, long tandem repeats (>70 bp) constituted 27% of all tandem repeats in the six *Ipomoea* cp genomes. *I. triloba* contained the longest repeat (484 bp). While most of the tandem repeats were conserved, a small number of specific tandem repeats were detected in the six *Ipomoea* cp genomes (**Figure 3D**). In particular, indel regions with different repeat copy numbers were identified for *I. nil* in *trnN-ycf1* (68 bp × one copy in *I. nil* / 68 bp × two copies in other *Ipomoea*) and *I. purpurea* in *ycf1* (18 bp × two copies in *I. purpurea* / 18 bp × three copies in other *Ipomoea*). These characteristics allowed indel markers for distinguishing *I. nil* and *I. purpurea* from other *Ipomoea* species to be developed in this study.

### Comparative Analysis of Six *Ipomoea* Chloroplast Genomes

**FIGURE 4:**
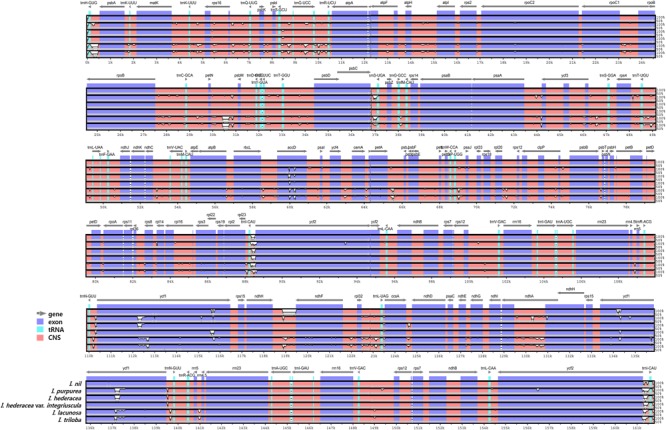
Comparison of six *Ipomoea* chloroplast genomes using mVISTA. Complete cp genomes of six *Ipomoea* species were compared, with *I. nil* as a reference. Blue block: conserved genes, sky-blue block: transfer RNA (tRNA) and ribosomal RNA (rRNA), and red block: conserved non-coding sequences (CNS). White represents regions with sequence variation among the six *Ipomoea* species.

**FIGURE 5:**
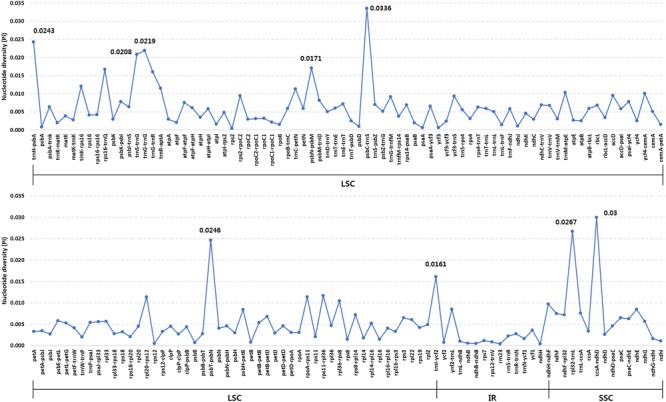
Comparison of nucleotide diversity (*Pi*) values among the six *Ipomoea* species.

Overall, the six *Ipomoea* cp genomes were highly conserved, with 98.5–99.8% similarity, conserved genomic structure, and conserved gene order and orientation (Supplementary Table S8). Pairwise determination of divergent regions was conducted using mVISTA (**Figure 4**). In general, non-coding regions were more diverged than coding regions. Five non-coding regions, *petN-psbM, trnI-CAU-ycf2, ndhH-ndhF, psbC-trnS*, and *ccsA-ndhD*, exhibited high divergence among the six *Ipomoea*. Coding regions were generally more conserved, with the exception of *ycf1, matK*, and *rbcL*, which are commonly used as representative plant DNA barcoding regions (CBOL Plant Working Group, 2009). Previous phylogenetic analysis of divergent non-coding regions allowed identification of potential molecular markers and DNA barcoding analysis (Shaw et al., 2007; Xu et al., 2017). *Pi* in the six *Ipomoea* cp genomes was calculated to show divergence at the sequence level (**Figure 5**). As expected, IR regions were more conserved than the LSC and SSC regions, with average *Pi* values of 0.003 for IR and 0.006 in SC (for regions other than those with a *Pi* value = 0). The average *Pi* value for coding regions was 0.00315 (range, 0.00038–0.00955; *accD* = 0.00955). The *Pi* value for intron-containing IGS averaged 0.00752 (range, 0.0005–0.00336; *psbC-trnS* in LSC = 0.00336). In the SSC, *ccsA-ndhD* exhibited a *Pi* value of 0.03, higher by an order of magnitude. Although the six *Ipomoea* cp genomes were generally highly conserved, the intergenic regions were particularly divergent. This is consistent with previous research with angiosperm cp genome (Ivanova et al., 2017). Similarly, the relatively higher divergence seen in the *ycf1, matK, rbcL*, and *accD* genes than in other coding regions was similar to observations in other cp genomes (Yukawa et al., 2006; Nie et al., 2012; Liu et al., 2013; Song et al., 2015).

IR regions were more highly conserved than SC regions due to copy correction by gene conversion in IR regions (Khakhlova and Bock, 2006). However, these events frequently lead to genome length variation through genome contraction and expansion (Raubeson et al., 2007), and IR contraction and expansion was previously used as an evolution criterion when examining cp genomes (Hansen et al., 2007; Huang et al., 2014). Here, we compared border regions and embedded genes among the six *Ipomoea* cp genomes (Supplementary Figure S2). The *rpl23* gene region in the six *Ipomoea* cp genomes was located in the LSC. All *trnH* genes were located in the LSC, 62–69 bp away from the IRb/LSC boundary. The *ndhH* genes were positioned in regions IRa and IRb. In particular, the *ndhA* gene extended into IRb. The *rpl23* gene shifted from the IR to the LSC, and *ndhH* exhibited gene duplication in IRs when compared with dicotyledon cp genomes (Yan et al., 2015). Although the genome structure of the IR region was highly conserved among the cp sequences of the six *Ipomoea* species, extreme gene shifting and duplication have occurred in the *Ipomoea* genus. To determine the selection pressure on protein-coding genes, we examined Ka/Ks (non-synonymous substitution to synonymous substitution) ratios from collinear genes as a marker of evolution (Supplementary Figure S3). Genes under positive selection are considered to be undergoing adaptive evolution in response to their environment (Kimura, 1989; Raman and Park, 2016; Ivanova et al., 2017). The most highly conserved genes exhibited purifying selection (Ka/Ks ratio, 0–0.001). The Ka/Ks ratios for most photosynthetic apparatus genes were close to 0. No significant gene evolution was observed according to regional groupings (i.e., LSC, IR, or SSC). Within the 6 *Ipomoea* cp genomes, 31 genes had Ka and Ks values >0.001, and the average Ka and Ks values were 0.0042 and 0.0168, respectively. The highest Ks value across the six *Ipomoea* species was 0.0717 (for *ndhE*), and the highest Ka/Ks ratio was 2.654 (for accD between *I. purpurea* and *I. lacunosa*). Thus, although the six *Ipomoea* cp genomes exhibited highly conserved organization, positive selection pressure (Ka/Ks > 1) was observed for *accD, cemA*, and *ycf2*. Positive selection of these three genes suggests that they are undergoing essential adaptations to their environment despite the weak selection pressures experienced by *Ipomoea*. Previous studies show that these genes are generally lost from, or are highly divergent in, angiosperms (Wicke et al., 2011; Ivanova et al., 2017). McNeal et al. (2007) showed that photosynthesis-related genes are under strong selection constraint in parasitic plants of the *Cuscuta* genus. Also, they reported that *accD, cemA*, and *ycf2* genes in both *Ipomoea* and *Cuscuta* were under purifying selection pressure. However, in the current study, we found that these genes were under positive selection pressure. Although plants within the *Cuscuta* genus express markedly diverse genes to adapt to life as parasitic plants, these genes might be rapidly evolving in the *Ipomoea* genus. Several studies show that nuclear genes in *Ipomoea* are under positive selection pressure. In particular, genes encoding dihydeoflavonol-4 reductase (DFR) and chalcone synthase (CHS) in *Ipomoea* are under positive selection pressure (Yang et al., 2004; Des Marais and Rausher, 2008). DFR is an important factor in the anthocyanin biosynthetic pathway. Des Marais and Rausher (2008) demonstrated that escape from adaptive conflict *via* repeated positive selection occurred after DFR genes duplicated in *I. purpurea*. Thus, DFR genes exhibit adaptive evolutionary changes. The CHS genes (which function in flavonoid biosynthesis) experienced selective pressure to promote divergence *via* increasing gene duplication in *Ipomoea. Ipomoea* has extremely diverse flower colors, a rapid generation time, and various growth forms. We suggest that these ecological characteristics of *Ipomoea* reflect their remarkable adaptability to various environments due to diverse positive selection pressure on genes in the nucleus or plastid.

### Phylogenic Relationships of Six *Ipomoea* Within Ipomoeeae

**FIGURE 6:**
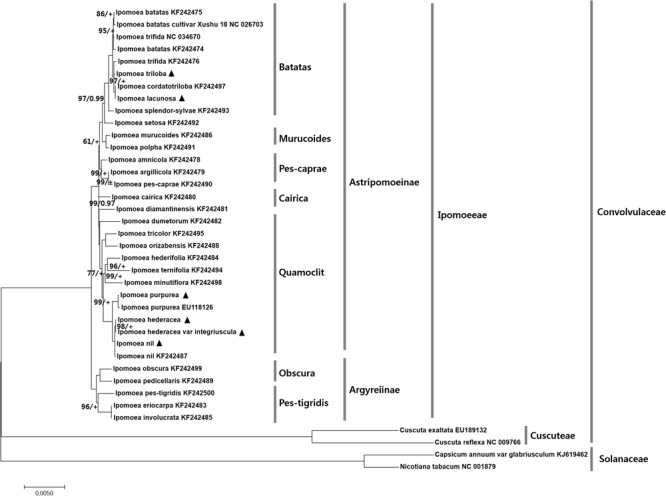
Phylogenetic tree based on 48 protein-coding genes from 6 *Ipomoea* and 28 other *Ipomoea* species using maximum likelihood (ML) bootstraps and Bayesian posterior probabilities (PP). ML topology is shown with ML bootstrap support values/ Bayesian PP given at each node. 100% bootstrap and 1.0 PP support are not marked. + Indicated 1.0 PP support values. Black triangles indicate the cp genomes of six *Ipomoea* species examined in this study.

Cp genomes are valuable genomic resources for reconstruction of accurate and high-resolution phylogenies, and have been used as such in several studies (Jansen et al., 2007; Moore et al., 2007), for example, in angiosperms (Wu et al., 2010; Nie et al., 2012). To identify the phylogenetic positions of the six *Ipomoea* species within the Convolvulaceae, we aligned 48 protein-coding sequences shared by 38 cp genomes (**Figure 6** and Supplementary Figure S4). The alignment length was 38,229 bp. All except two nodes were supported by a Bayesian PP of 1.0. *Ipomoea* and *Cuscuta* had the closest phylogenetic relationship within the Convolvulaceae. Consistent with previous analysis, the tribe Ipomoeeae was divided into 2 major clades, Atstipomoeinae and Atgyreiinae, with 28 *Ipomoea* species within 7 sections (Stefanoviæ et al., 2003). Most *Ipomoea* species were within Quamoclit and Batatas. The positions of the six *Ipomoea* examined in this study were strongly supported with BI and PP values. *I. nil* and *I. hederacea* formed a monophyletic cluster as a sister to *I. purpurea* within Quamoclit *I. lacunosa* and *I. triloba* formed a monophyletic cluster in Batatas. Previous analysis of 28 *Ipomoea* cp genomes clarified the evolutionary relationships within the 2 major clades of Ipomoeeae (Eserman et al., 2014). Quamoclit species were divided into two clades (Miller et al., 2004), whereas our phylogenetic results revealed their monophyly. In this study, *I. hederacea* and *I. hederacea* var. *integriuscula* were clustered with *I. nil* in a monophyletic relationship, but that *I. purpurea* was paraphyletic with these species. A previous study indicated that *I. nil* and *I. purpurea* share a monophyletic relationship. Here, we obtained more accurate information about the relationship between *I. nil* and *I. hederacea* and *I. hederacea* var. *integriuscula* and *I. purpurea*. Furthermore, *I. triloba* and *I. lacunosa* were positioned in Batatas as a monophyletic group with *I. trifida* and *I. cordatotriloba*. Therefore, we performed high-resolution phylogenetic analysis of the positions of *Ipomoea* species in Batatas in the phylogenic tree. The reconstructed phylogenic trees were clearly consistent with previous studies according to the APG IV system (Austin, 1978; Eserman et al., 2014; The Angiosperm Phylogeny Group, 2016). The results of this study are strongly supported by those of previous studies; however, we further clarified the phylogenetic relationships within the Ipomoeeae. Based on the phylogenetic positions of the six *Ipomoea* species determined in the present study, *I. nil* and *I. purpurea* (whose seeds are used for Pharbitidis Semen) share closer relationships with *I. hederacea* and *I. hederacea* var. *integriuscula* than with *I. triloba* and *I. lacunosa*. Thus, there is a strong possibility for confusion between *I. hederacea* and *I. hederacea* var. *integriuscula*. We suspect that the most frequent adulterations of Pharbitidis Semen are seeds of *I. hederacea* and *I. hederacea* var. *integriuscula*.

### New Indel Markers for Distinguishing Herbal Medicine Plants

**FIGURE 7:**
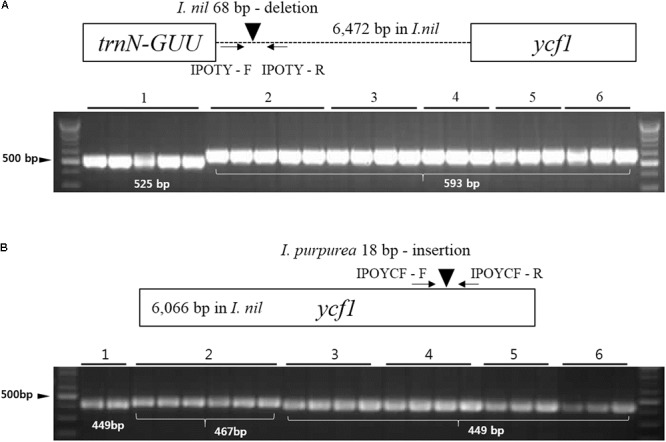
Schematic of indel markers IPOTY and IPOYCF for *I. nil* and *I. purpurea*. **(A)** Primers for IPOTY were tested with 23 *Ipomoea* germplasms. **(B)** Primers for IPOYCF were tested with 22 *Ipomoea* germplasms. Germplasm details are provided in Supplementary Table S1. 1, *Ipomoea nil*; 2, *I. purpurea*; 3, *I. hederacea*; 4, *I. hederacea* var. *integriuscula*; 5, *I. lacunosa*; and 6, *I. triloba*.

Dried seeds of *I. nil* and *I. purpurea* are used in traditional herbal medicine in Korea (Korea Institute of Oriental Medicine [KIOM], 2016). However, seeds from other *Ipomoea* strongly resemble those of *I. nil* and *I. purpurea* and are often inappropriately included in herbal preparations. Although the phylogenetic analysis in this study indicated that *I. triloba* and *I. lacunosa* were phylogenetically distant from *I. nil* and *I. purpurea*, the highly similar seed shapes present a challenge for identifying authentic herbal medicines, and a molecular approach would be beneficial. Here, DNA barcode analysis was performed for *I. nil* and *I. purpurea* and four related *Ipomoea* species, *I. triloba, I. lacunosa, I. hederacea*, and *I. hederacea* var. *integriuscula*, with similar seed shapes. *I. purpurea* was distinguished from other *Ipomoea* species with the ITS2 and *matK* regions (Supplementary Figure S5). However, the sequence of *I. nil* at ITS2 was the same as that of *I. hederacea*, and the sequence of *I. nil* at *matK* was the same as that of *I. lacunosa* and *I. triloba*, highlighting the limitations of universal DNA barcode sequences for distinguishing species. To resolve this problem, divergent regions within the cp genome were examined with the aim of distinguishing *I. nil* and *I. purpurea*. This analysis revealed species-specific divergent regions at *trnN-ycf1* and *ycf1* for *I. nil* and *I. purpurea*, respectively, with respect to copy number variation in tandem repeats. To develop indel markers, specific primers were designed against conserved regions of *trnN-ycf1* and *ycf1* (**Table 3**). The primer pairs, respectively named IPOTY and IPOYCF, successfully amplified sequences from *I. nil* and *I. purpurea* (**Figure 7**). The markers were tested with other *Ipomoea* germplasms (23 samples for IPOTY and 22 samples for IPOYCF), and the five *I. nil* samples and six *I. purpurea* samples were clearly distinguishable. Amplified fragments from all of the tested *Ipomoea* samples were sequenced to identify exact amplicon size. IPOTY primers yielded a 525 bp amplicon with *I. nil*, and IPOYCF primers yielded a 467 bp fragment with *I. purpurea*. Predicted deletion or insertion sizes from cp genomes were consistent with those resulting from the *Ipomoea* germplasms used in this study. Indel markers for variable copy numbers at tandem repeats were also used previously to distinguish closely related *Fagopyrum* and *Chenopodium* species, indicating the utility of these markers in species identification (Cho et al., 2015; Hong et al., 2017). Copy number variation at tandem repeats in cp genomes may, therefore, prove broadly useful in distinguishing closely related plant species where universal barcode sequences are non-discriminatory. The IPOTY and IPYCF indel markers developed in this study will be useful for *Ipomoea* species identification and authentication of herbal medicines.

**Table 3:** Primer information for indel markers IPOTY and IPOYCF.

Primer name	Primer sequence (5′ > 3′)	Position
IPOTY_F	TAACGGTCAAAGCGAGCCCC	*trnN-GUU–ycf1*
IPOTY_R	AAGTCCAGCCGCAAGAACTGA	
IPOYCF_F	GGTCGCGGTAAATCCCAGCA	*ycf1*
IPOYCF_R	TCTTCCCAGAATTTGTGCGGC	

## Conclusion

Six *Ipomoea* cp genomes were sequenced in this study. Overall, the cp genomes were highly conserved with respect to gene content, gene orientation, and GC content, but local variations in sequence and structure were observed. Tandem repeats and SSRs were identified with the aim of developing molecular markers for species identification and authentication of herbal medicines. The most divergent regions among the six genomes were found in non-coding regions *petN-psbM, trnI-CAU-ycf2, ndhH-ndhF, psbC-trnS*, and *ccsA-ndhD*, and coding regions *accD, cemA*, and *ycf2.* The *accD, cemA*, and *ycf2* genes exhibited positive selection. Phylogenetic analysis of cp genome sequences yielded more accurate phylogenetic relationships within the *Ipomoea* genus than previous studies. Novel indel markers based on copy number variation at tandem repeats were developed for identification of *I. nil* and *I. purpurea*. These markers, named IPOTY and IPOYCF, were able to discriminate between authentic *I. nil* and *I. purpurea* and other inauthentic *Ipomoea* species, respectively, and will be useful for authentication of herbal medicines containing these two species. The cp genomes and analyses in this study are valuable for species identification, clarification of taxonomy, and understanding evolutionary history in the *Ipomoea* genus.

## Author Contributions

IP designed the experimental framework and drafted and revised the manuscript. SY and BCM collected and identified plant materials. WJK and PN performed the experiments. HOL carried out sequence analysis. BCM revised the manuscript. All authors contributed to the experiments and approved the final manuscript.

## Conflict of Interest Statement

The authors declare that the research was conducted in the absence of any commercial or financial relationships that could be construed as a potential conflict of interest. The reviewer AZ and the handling Editor declared their shared affiliation.
