# Supplementary material for: The Complete Chloroplast Genomes of Six Ipomoea Species and Indel Marker Development for the Discrimination of Authentic Pharbitidis Semen (Seeds of I. nil or I. purpurea)
Source: Front Plant Sci. 2018 Jul 5;9:965. doi: 10.3389/fpls.2018.00965 (PMC6041466; doi:10.3389/fpls.2018.00965)
Supplement: Supplementary file 1 [file Image_1.pdf]

Supplementary Material

The Complete Chloroplast Genomes of Six *Ipomoea* Species and Indel Marker Development for the Discrimination of Authentic Pharbitidis Semen (seeds of *I. nil* or *I. purpurea*)

Inkyu Park, Sungyu Yang, Wook Jin Kim, Pureum Noh, Hyun Oh Lee, Byeong Cheol Moon*

***Correspondence:** Byeong-Cheol Moon: bcmoon@kiom.re.kr

Supplementary Figures and Tables

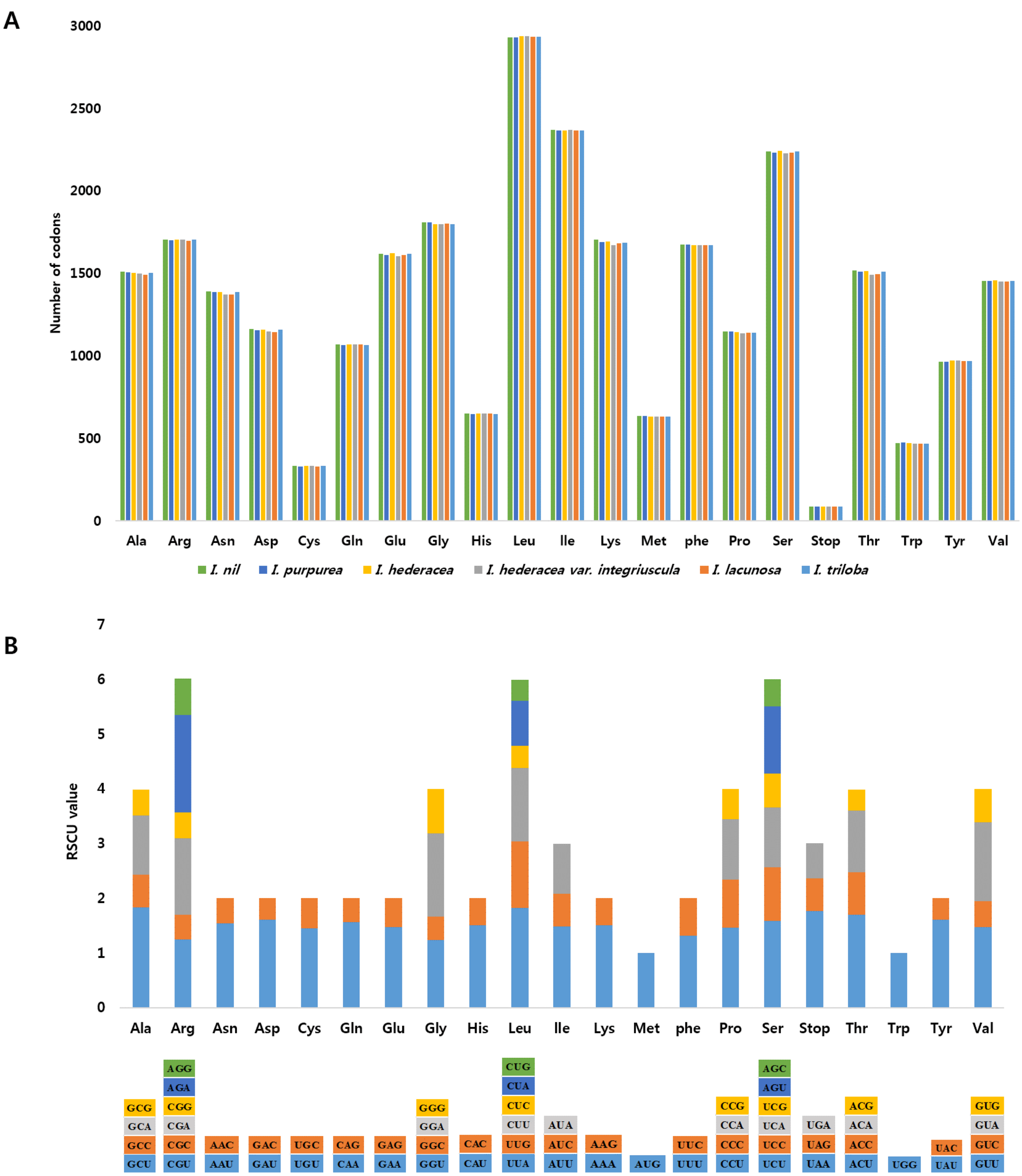

Figure S1 Codon frequencies and RSCU values for six *Ipomoea* chloroplast genomes. (A) Amino acid frequencies for protein-coding sequences. (B) Codon usage for 20 amino acids and stop codons in 78 protein-coding genes.

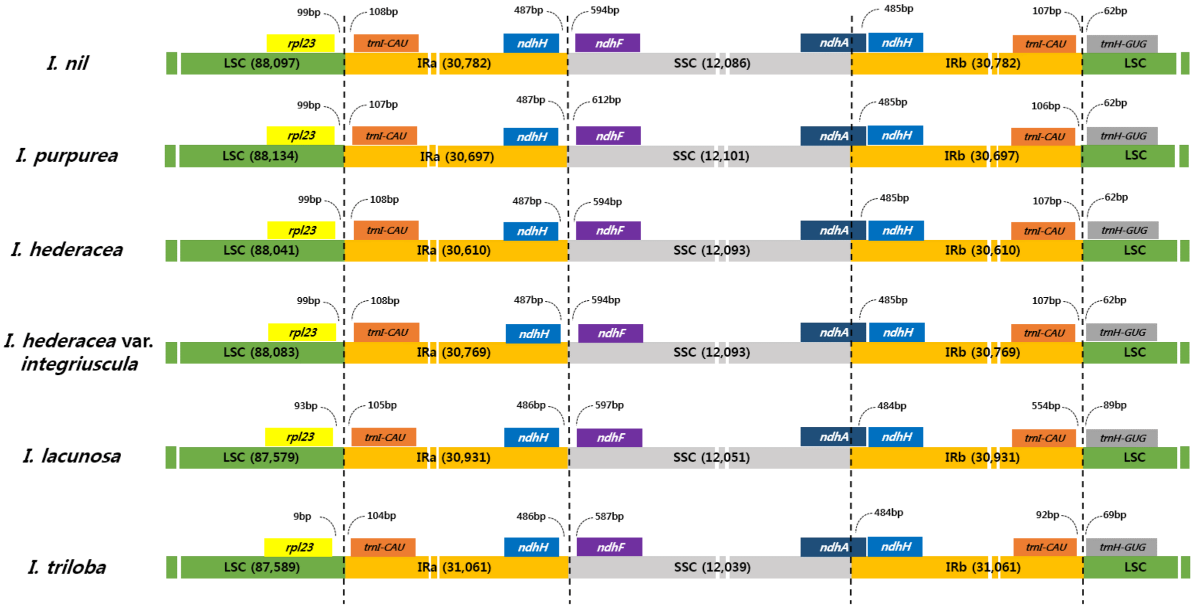

Figure S2 Comparison of the LSC, IR, and SSC junction positions in six *Ipomoea* chloroplast genomes.

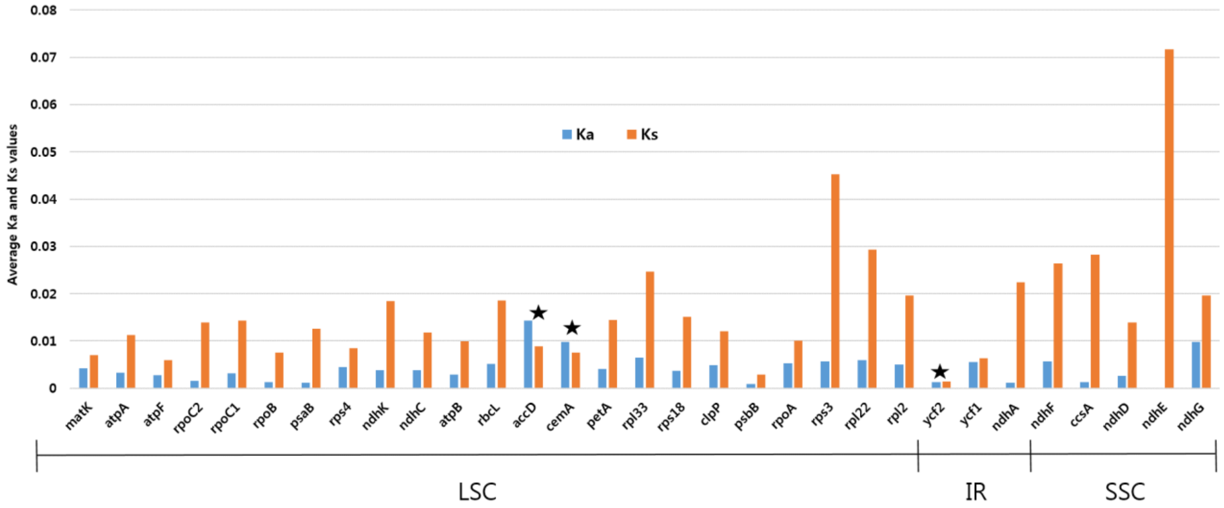

Figure S3 Ka and Ks values for six *Ipomoea* cp genomes. The ratios of non-synonymous substitution (Ka) to synonymous substitution (Ks) were calculated for 78 conserved protein-coding sequences. Genes with Ka or Ks = 0 are not shown. Average Ka and Ks values are shown in blue and orange, respectively. Black stars indicate genes undergoing positive selection (Ka/Ks >1) among the six *Ipomoea* species.

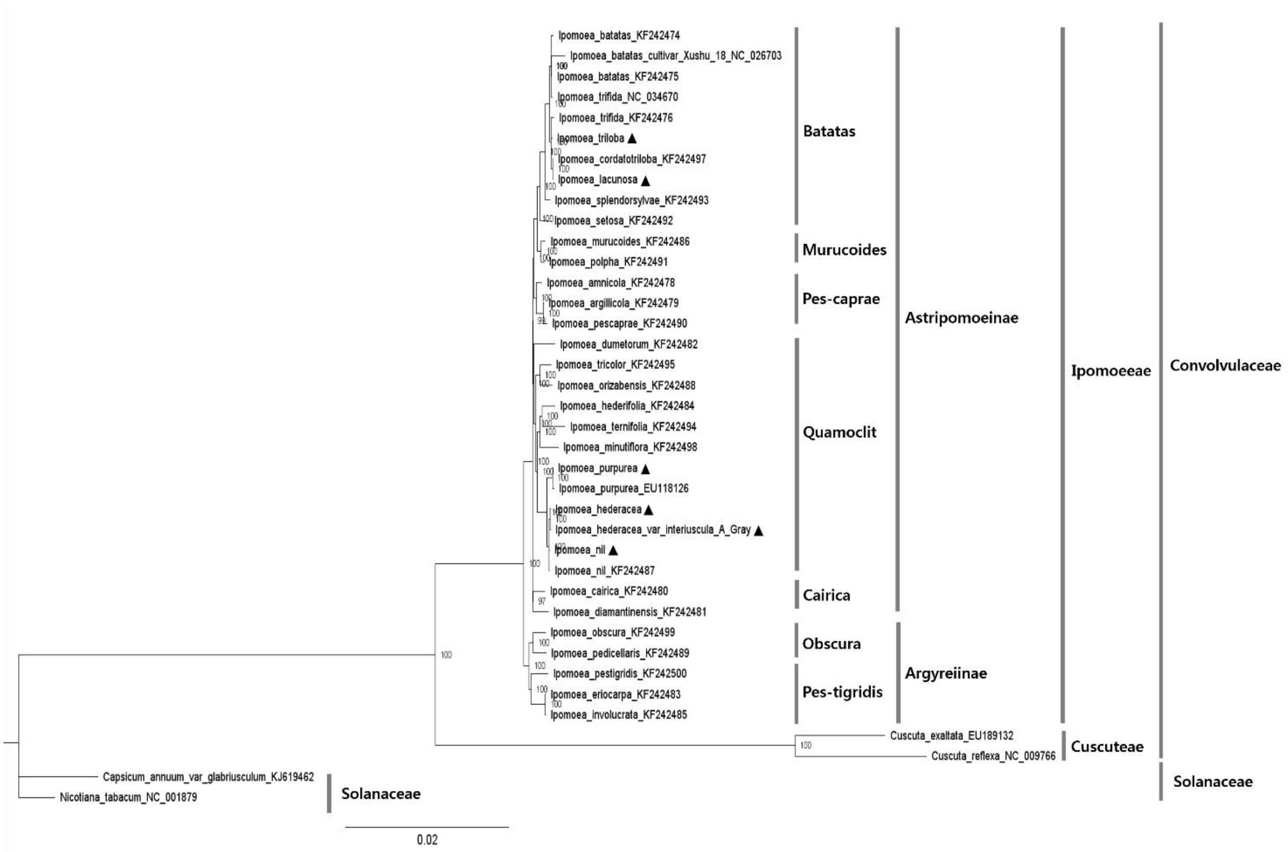

Figure S4 Phylogenetic tree based on 48 protein-coding genes from six *Ipomoea* and 28 *Ipomoea* species using Bayesian posterior probabilities (PP).

ITS2

Nucleotide-position	117	133	156	190	380-382	435	460	470	498	519	537	556	558-559	560	569
I. nil	**C**	**G**	**T**	**C**	**CCT**	**C**	**T**	**C**	**C**	**C**	**G**	**C**	**GT**	**C**	**C**
I. purpurea	C	C	T	T	GAG	T	C	T	T	G	A	T	AG	C	T
I. hederacea	C	G	T	C	CCT	C	T	C	C	C	G	C	GT	C	C
I. hederacea var. *integriuscula*	C	G	T	C	CCT	C	T	C	C	C	G	C	GT	C	C
I. lacunosa	A	G	C	C	CCT	C	T	C	C	C	G	C	GT	T	C
I. triloba	T	G	G	C	CCT	C	T	C	C	C	G	C	GT	C	C

matK

Nucleotide-position	1155	1157-1158
I. nil	**G**	**GT**
I. purpurea	G	AC
I. hederacea	A	GT
I. hederacea var. *integriuscula*	A	GT
I. lacunosa	G	GT
I. triloba	G	GT

Figure S5 Schematic diagram showing DNA barcode analysis of six *Ipomoea* species using universal DNA barcode primers for ITS2 (ITS-s2f, ITS4) and *matK* (matK-AF, matK-8R).

Table S1 Germplasms used in this study

No.	Species	Collection information	Voucher number	Completed chloroplast genome	IPOTY test	IPO-YCF1 test
1	*I. nil*	Namyang-myeon, Goheung-gun, Jeollanam-do, Korea	KIOM201701018913	o	o	o
2		Hallim-eup, Jeju-si, Jeju, Korea	KIOM201601017919		o	o
3		Jeonmin-dong, Yuseong-gu, Daejeon, Korea	KIOM-2017-78		o	
4		Gujeuk-dong, Yuseong-gu, Daejeon, Korea	KIOM-2016-333		o	
5		Dongi-myeon, Okcheon-gun, Chungcheongbuk-do, Korea	KIOM201501012234		o	
6	*I. purpurea*	Namyang-myeon, Goheung-gun, Jeollanam-do, Korea	KIOM201701018916	o	o	o
5		Dongseo-dong, Sacheon-si, Gyeongsangnam-do, Korea	KIOM201601018515		o	o
6		Jasan-ri, Donghyang-myeon, Jinan-gun, Jeollabuk-do, Korea	KIOM201701018871		o	o
7		Bongsan-dong, Yuseong-gu, Daejeon, Korea	KIOM201701018883		o	o
8		Jugwang-myeon, Goseong-gun, Gangwon-do, Korea	KIOM-2016-315		o	o
9		Boan-myeon, Buan-gun, Jeollabuk-do, Korea	KIOM201701018893			o
10	*I. hederacea*	Namyang-myeon, Goheung-gun, Jeollanam-do, Korea	KIOM201701018903	o	o	o
11		Gwanpyeong-dong, Yuseong-gu, Daejeon, Korea	KIOM201601018486		o	o
12		Ochang-eup, Cheongju-si, Chungcheongbuk-do, Korea	KIOM201701018855		o	o
13		Gonyang-myeon, Sacheon-si, Gyeongsangnam-do, Korea	KIOM-2016-322		o	o
14	*I. hederacea* var. *integriuscula*	Namyang-myeon, Goheung-gun, Jeollanam-do, Korea	KIOM201701018905	o	o	o
15		Hallim-eup, Jeju-si, Jeju, Korea	KIOM201601017921		o	o
16		Hajodae, Yangyang-gun, Gangwon-do, Korea	KIOM-2016-317		o	o
17		Gahoe-myeon, Hapcheon-gun, Gyeongsangnam-do, Korea	KIOM-2016-331			o
18	*I. lacunosa*	Namyang-myeon, Goheung-gun, Jeollanam-do, Korea	KIOM201701018906	o	o	o
19		Gahoe-myeon, Hapcheon-gun, Gyeongsangnam-do, Korea	KIOM-2016-324		o	o
20		Boan-myeon, Buan-gun, Jeollabuk-do, Korea	KIOM201701018899		o	o
21	*I. triloba*	Joseong-myeon, Boseong-gun, Jeollanam-do, Korea	KIOM201701018921	o	o	o
22		Joseong-myeon, Boseong-gun, Jeollanam-do, Korea	KIOM201701018922		o	o
23		Joseong-myeon, Boseong-gun, Jeollanam-do, Korea	KIOM201701018923		o	o

Table S2 Primers used in this study for CP junction validation

Primer name	Primer sequence (5`>3`)	Position
IPOLI1F	GGATGAGATTCCAAGGTCCTGATT	LSC_IRa
IPOLI1R	GTGGACAAGGGTCCCTGTTA	
IPOIS1F	CGCCGCTTCAAGTATTGCTC	IRa_SSC
IPOIS1R	CCTTTTCTGCCTCAAGTGCG	
IPOSI1F	TCTTTGAAACCCGCTTCCGA	SSC_IRb
IPOSI1R	CGCCGCTTCAAGTATTGCTC	
IPOIL1F	CATCCATGGCTGAGTGGTGA	IRb_LSC

IPOIL1R	CTATGCGCGGGTTCAATTCC

Table S3 PCR-based sequence validation of cp junctions

Species	location	PCR-based sequence (bp)	CP sequence (bp)	start	end	Identities	%
I. nil	LSC_IRa	122	161,747	88,177	88,298	122/122	100
	IRa_SSC	188		118,805	118,992	188/188	100
	SSC_IRb	814		130,227	131,040	814/814	100
	IRb_LSC	271		161,569	92	271/271	100
I. purpurea	LSC_IRa	122	161,629	88,213	88,334	122/122	100
	IRa_SSC	206		118,757	118,962	206/206	100
	SSC_IRb	812		130,196	131,007	812/812	100
	IRb_LSC	270		161,452	92	270/270	100
I. hederacea	LSC_IRa	122	161,354	88,121	88,242	122/122	100
	IRa_SSC	188		118,577	118,764	188/188	100
	SSC_IRb	814		130,006	130,819	814/814	100
	IRb_LSC	271		161,176	92	271/271	100
I. hederacea var. *integriuscula*	LSC_IRa	122	161,714	88,163	88,284	122/122	100
	IRa_SSC	188		118,778	118,965	188/188	100
	SSC_IRb	814		130,207	131,020	814/814	100
	IRb_LSC	271		161,536	92	271/271	100
I. lacunosa	LSC_IRa	122	161,492	87,656	87,777	122/122	100
	IRa_SSC	188		118,437	118,610	188/188	100
	SSC_IRb	793		129,843	130,635	793/793	100
	IRb_LSC	294		161,317	119	294/294	100
I. triloba	LSC_IRa	122	161,750	87,654	87,775	122/122	100
	IRa_SSC	188		118,577	118,750	188/188	100
	SSC_IRb	791		129,973	130,763	791/791	100
	IRb_LSC	263		161,587	99	263/263	100

Table S4 Chloroplast genomes from NCBI used for phylogenetic analysis

No.	Family	Taxon	GenBank accession number
1	Convolvulaceae	*Ipomoea purpurea*	EU118126.1
2		*Ipomoea batatas cultivar Xushu 18*	NC_026703.1
3		*Ipomoea pes-tigridis*	KF242500.1
4		*Ipomoea obscura*	KF242499.1
5		*Ipomoea minutiflora*	KF242498.1
6		*Ipomoea tricolor*	KF242495.1
7		*Ipomoea ternifolia*	KF242494.1
8		*Ipomoea setosa*	KF242492.1
9		*Ipomoea polpha*	KF242491.1
10		*Ipomoea pes-caprae*	KF242490.1
11		*Ipomoea pedicellaris*	KF242489.1
12		*Ipomoea orizabensis*	KF242488.1
13		*Ipomoea nil*	KF242487.1
14		*Ipomoea murucoides*	KF242486.1
15		*Ipomoea involucrata*	KF242485.1
16		*Ipomoea hederifolia*	KF242484.1
17		*Ipomoea eriocarpa*	KF242483.1
18		*Ipomoea dumetorum*	KF242482.1
19		*Ipomoea diamantinensis*	KF242481.1
20		*Ipomoea cairica*	KF242480.1
21		*Ipomoea argillicola*	KF242479.1
22		*Ipomoea amnicola*	KF242478.1
23		*Ipomoea splendor-sylvae*	KF242493.1
24		*Ipomoea trifida*	KF242476.1
25		*Ipomoea batatas*	KF242475.1
26		*Ipomoea batatas*	KF242474.1
27		*Ipomoea cordatotriloba*	KF242497.1
28		*Ipomoea trifida*	NC_034670.1
29		*Cuscuta exaltata*	EU189132.1
30		*Cuscuta reflexa*	NC_009766.1
31	Solanaceae	*Nicotiana tabacum*	NC_001879.2
32		*Capsicum annuum* var. glabriusculum	KJ619462.1

Table S5 Raw reads and trimmed reads data

Scientific name	Input reads	Trimmed reads		Total raw bases	Trimmed bases	
Ipomoea nil	4,573,202	3,628,472	79.34%	1,358,210,596	843,982,036	62.14%
Ipomoea purpurea	5,070,256	4,127,530	81.41%	1,496,288,384	962,181,758	64.30%

Ipomoea hederacea	5,464,740	4,340,128	79.42%	1,624,707,998	1,011,823,016	62.28%
Ipomoea hederacea var. *integriuscula*	4,578,130	3,637,141	79.45%	1,359,746,277	847,364,988	62.32%
Ipomoea lacunosa	4,564,630	3,661,941	80.22%	1,355,877,695	857,378,293	63.23%
Ipomoea triloba	4,452,482	3,455,730	77.61%	1,323,463,129	799,351,956	60.40%

Table S6 Genome assembly information for six *Ipomoea* chloroplast genomes

Scientific name	Aligned reads (#)	Coverage (x)	Cp genome length (bp)
Ipomoea nil	310,901	443.10	161,747
Ipomoea purpurea	273,240	391.36	161,629
Ipomoea hederacea	427,231	611.58	161,354
Ipomoea hederacea var. *integriuscula*	269,581	384.78	161,714
Ipomoea lacunosa	352,038	503.72	161,492
Ipomoea triloba	277,309	390.00	161,750

Table S7 Genic introns in six *Ipomoea* chloroplast genomes

I. nil	Gene	region	exon I	intron I	exon II	intron II	exon III
1	trnk-UUU	LSC	37	2548	35		
2	rps16	LSC	40	805	221		
3	trnG-UCC	LSC	23	860	37		
4	atpF	LSC	144	699	411		
5	rpoC1	LSC	432	813	1611		
6	ycf3	LSC	126	726	228	770	153
7	trnL-UAA	LSC	35	361	34		
8	trnV-UAC	LSC	38	586	37		
9	rps12	LSC	114		232		26
10	ClpP	LSC	71	803	292	614	252
11	petB	LSC	6	755	642		
12	petD	LSC	8	701	475		
13	rpl16	LSC	9	1025	435		
14	ndhB	IR	777	673	750		
15	trnI-GAU	IR	42	927	35		
16	trnA-UGC	IR	38	813	35		
17	ndhA	SSC	557	1443	541		
I. purpurea	Gene	region	exon I	intron I	exon II	intron II	exon III
1	trnk-UUU	LSC	37	2563	35		
2	rps16	LSC	40	812	221		
3	trnG-UCC	LSC	23	871	48		
4	atpF	LSC	144	698	411		
5	rpoC1	LSC	432	812	1611		
6	ycf3	LSC	126	726	228	770	153
7	trnL-UAA	LSC	35	362	34		
8	trnV-UAC	LSC	38	586	37		
9	rps12	LSC	114		232		26
10	ClpP	LSC	71	810	292	612	252
11	petB	LSC	6	755	642		
12	petD	LSC	8	702	475		
13	rpl16	LSC	9	1019	435		
14	ndhB	IR	777	673	750		
15	trnI-GAU	IR	42	927	35		
16	trnA-UGC	IR	38	813	35		
17	ndhA	SSC	557	1432	541		
I. hederacea	Gene	region	exon I	intron I	exon II	intron II	exon III
1	trnk-UUU	LSC	37	2548	35		
2	rps16	LSC	40	810	221		
3	trnG-UCC	LSC	23	860	48		
4	atpF	LSC	144	698	411		
5	rpoC1	LSC	432	812	1611		
6	ycf3	LSC	126	726	228	770	153
7	trnL-UAA	LSC	35	361	34		
8	trnV-UAC	LSC	38	586	37		

9	rps12	LSC	114		232		26
10	ClpP	LSC	71	803	292	615	252
11	petB	LSC	6	755	642		
12	petD	LSC	8	701	475		
13	rpl16	LSC	9	1025	435		
14	ndhB	IR	777	673	750		
15	trnI-GAU	IR	42	927	35		
16	trnA-UGC	IR	38	813	35		
17	ndhA	SSC	557	1443	541		
I. hederacea var. *integriuscula*	Gene	region	exon I	intron I	exon II	intron II	exon III
1	trnk-UUU	LSC	37	2548	35		
2	rps16	LSC	40	810	221		
3	trnG-UCC	LSC	23	860	48		
4	atpF	LSC	144	698	411		
5	rpoC1	LSC	432	812	1611		
6	ycf3	LSC	126	726	228	770	153
7	trnL-UAA	LSC	35	361	34		
8	trnV-UAC	LSC	38	586	37		
9	rps12	LSC	114		232		26
10	ClpP	LSC	71	803	295	612	252
11	petB	LSC	6	755	642		
12	petD	LSC	8	701	475		
13	rpl16	LSC	9	1025	435		
14	ndhB	IR	777	673	750		
15	trnI-GAU	IR	42	927	35		
16	trnA-UGC	IR	38	813	35		
17	ndhA	IR	557	1443	541		
I. lacunosa	Gene	region	exon I	intron I	exon II	intron II	exon III
1	trnk-UUU	LSC	37	2526	35		
2	rps16	LSC	40	819	221		
3	trnG-UCC	LSC	23	873	48		
4	atpF	LSC	144	703	411		
5	rpoC1	LSC	432	829	1611		
6	ycf3	LSC	126	717	228	770	153
7	trnL-UAA	LSC	35	361	34		
8	trnV-UAC	LSC	38	586	37		
9	rps12	LSC	114		232		26
10	ClpP	LSC	71	793	295	608	252
11	petB	LSC	6	756	642		
12	petD	LSC	8	706	475		
13	rpl16	LSC	9	1023	435		
14	ndhB	IR	777	673	750		
15	trnI-GAU	IR	42	927	35		
16	trnA-UGC	IR	38	813	35		
17	ndhA	IR	557	1428	541		
I. triloba	Gene	region	exon I	intron I	exon II	intron II	exon III
1	trnk-UUU	LSC	37	2535	35		

2	rps16	LSC	40	819	221		
3	trnG-UCC	LSC	23	873	48		
4	atpF	LSC	144	702	411		
5	rpoC1	LSC	432	828	1611		
6	ycf3	LSC	126	717	228	770	153
7	trnL-UAA	LSC	34	361	34		
8	trnV-UAC	LSC	38	586	37		
9	rps12	LSC	114		232		26
10	ClpP	LSC	71	793	292	612	252
11	petB	LSC	6	756	642		
12	petD	LSC	8	701	475		
13	rpl16	LSC	9	1022	435		
14	ndhB	IR	777	673	750		
15	trnI-GAU	IR	42	927	35		
16	trnA-UGC	IR	38	813	35		
17	ndhA	IR	557	1426	541		

Table S8 Similarities between the chloroplast genomes of six *Ipomoea* species

**Similarity**	***I. nil***	***I. purpurea***	***I. hederacea***	***I. hederacea* var. *interiuscula***	***I. lacunosa***	***I. triloba***
I. nil	-	99.45%	99.85%	99.82%	98.82%	98.68%
I. purpurea		-	99.57%	99.44%	98.61%	98.52%
I. hederacea			-	99.77%	98.62%	98.50%
I. hederacea var. *integriuscula*				-	98.81%	98.69%
I. lacunosa					-	99.72%
I. triloba						-
